# Multi-omics Characterization of Interaction-mediated Control of Human Protein Abundance levels*[Fn FN2]

**DOI:** 10.1074/mcp.RA118.001280

**Published:** 2019-06-25

**Authors:** Abel Sousa, Emanuel Gonçalves, Bogdan Mirauta, David Ochoa, Oliver Stegle, Pedro Beltrao

**Affiliations:** ‡Instituto de Investigação e Inovação em Saúde da Universidade do Porto (i3s), Rua Alfredo Allen 208, 4200–135, Porto, Portugal; §Institute of Molecular Pathology and Immunology of the University of Porto (IPATIMUP), Rua Júlio Amaral de Carvalho 45, 4200–135, Porto, Portugal; ¶Graduate Program in Areas of Basic and Applied Biology (GABBA), Abel Salazar Biomedical Sciences Institute, University of Porto, Rua de Jorge Viterbo Ferreira 228, 4050–313, Porto, Portugal; ‖European Molecular Biology Laboratory, European Bioinformatics Institute, Wellcome Genome Campus, Hinxton, CB10 1SD, Cambridge, UK; **Wellcome Sanger Institute, Wellcome Genome Campus, Hinxton, UK; ‡‡European Molecular Biology Laboratory, Genome Biology Unit, 69117 Heidelberg, Germany; §§Division of Computational Genomics and Systems Genetics, German Cancer Research Center (DKFZ), 69120, Heidelberg, Germany

**Keywords:** Proteogenomics, Cancer Biology, Bioinformatics, Computational Biology, Posttranslational Modifications

## Abstract

Proteogenomic studies of cancer samples have shown that copy-number variation can be attenuated at the protein level for a large fraction of the proteome, likely due to the degradation of unassembled protein complex subunits. Such interaction-mediated control of protein abundance remains poorly characterized. To study this, we compiled genomic, (phospho)proteomic and structural data for hundreds of cancer samples and find that up to 42% of 8,124 analyzed proteins show signs of post-transcriptional control. We find evidence of interaction-dependent control of protein abundance, correlated with interface size, for 516 protein pairs, with some interactions further controlled by phosphorylation. Finally, these findings in cancer were reflected in variation in protein levels in normal tissues. Importantly, expression differences due to natural genetic variation were increasingly buffered from phenotype differences for highly attenuated proteins. Altogether, this study further highlights the importance of posttranscriptional control of protein abundance in cancer and healthy cells.

## Abstract (precis)

The degree and mechanisms by which gene copy-number changes are buffered at the protein level are not understood. We have identified up to 40% of genes with protein-level buffering of gene dosage changes in cancer. Using structural data, we show that interaction-dependent control of abundance is correlated with interface size. These findings in cancer were reflected in variation in protein levels in normal tissues with buffering of natural genetic variation for highly attenuated proteins.

## Graphical Abstract

**Figure:**
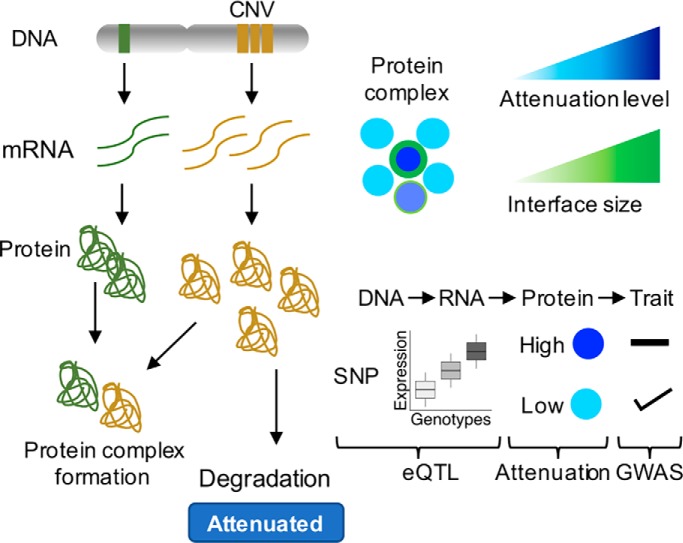

- Up to ∼40% of proteins have copy-number changes attenuated, likely via degradation.
- Interaction-dependent protein attenuations correlates with interface size.
- Protein attenuation is reflected in variation in protein levels in normal tissues.
- Protein attenuation buffers expression differences due to natural genetic variation.

Cancer cells can harbor a large number of somatic DNA alterations ranging from point mutations to gene copy changes that can occur from deletion or amplification of small regions or whole chromosomes. While these events are the source of the genetic variation that can confer a selective advantage and lead to cancer, large changes in gene numbers can be detrimental and cause imbalances in the corresponding protein levels. Several studies have shown that the majority of changes in gene copy number will propagate to changes in the corresponding protein levels (1–3). However, models of aneuploidy of different species and analysis of gene copy-number variation (CNV) in cancer have shown that CNVs of protein-coding genes belonging to protein complexes tend to be attenuated at the protein level (1, 4, 5). In addition, we have shown that some complex members can act as rate-limiting subunits and indirectly control the degradation level of attenuated complex members (4). These results are in-line with pulse-chase degradation measurements showing that several complex subunits have a two-state degradation profile that is compatible with a model in which they are expressed above the required levels and have a higher degradation rate when unbound from the complex (6). The attenuation of changes at the protein level also justifies why protein complex subunits show higher correlation of protein abundances than the corresponding mRNA levels (7, 8) and why correlation analysis can be used to identify cancer-specific interaction networks (9, 10).

These results support a long-standing view that protein complex formation can set the total amount of protein levels (11). The degradation of unbound subunits may be due to a requirement of avoiding free hydrophobic interface surfaces that can be prone to aggregate (12). In eukaryotic species, this appears to be achieved by degrading excess production, while in bacterial and archaeal species genes coding for protein complexes subunits tend to occur within operon structures such that they will be expressed at similar levels (13). This link between appropriate expression and complex formation is further emphasized by the preferential ordering of subunits in operons starting from the subunits that tend to assemble first (14).

While this phenomenon of gene dosage attenuation in protein complexes has been well documented, we still do not understand (i) what protein properties are associated with the propensity for a protein to be attenuated or (ii) if the characteristics of the attenuation process are seen in noncancerous cells. Here we have extended on a previous analysis (4), performing a multi-omics study of protein-level attenuation of gene dosage that combines genomics, (phospho)proteomics, and structural data. Analyzing 8,124 genes/proteins we observed that up to 42% of proteins show evidence of posttranscriptional regulation. Over 500 protein–protein interactions show indirect control of degradation of one subunit via physical associations, 32 of which may be further controlled by phosphorylation. Using structural models for 3,082 interfaces, we find that a higher fraction of interface residues is associated with a higher degree of attenuation. Finally, we studied the impact of these findings on noncancerous systems. We find that protein interaction-mediated control of protein abundances have an impact of the variation of protein levels across tissues and that the degree of attenuation correlates with the probability that natural variation with an impact on gene expression may result in a phenotypic consequence.

## EXPERIMENTAL PROCEDURES

### 

#### 

##### Multi-omics Data Collection

Proteomics and phosphoproteomics quantifications at the protein/phosphosite level from TCGA cancer patients were obtained from the CPTAC data portal (proteomics.cancer.gov/data-portal), for breast cancer (BRCA) (15), colorectal cancer (COREAD) (16), and ovarian cancer (17). The same data from cancer cell lines were downloaded for COREAD cell lines (10) and for BRCA cell lines (9, 18). Gene-level RNA-seq raw counts were acquired from gene expression omnibus (GSE62944) (19) for TCGA samples and from the CCLE data portal (19–21) for cancer cell lines. Gene copy-number profiles in this study were represented using discretized GISTIC 2.0 scores as described here (22, 23). Briefly, these discrete variables can be −2 (strong copy-number loss, likely a homozygous deletion), −1 (shallow deletion, likely a heterozygous deletion), 0 (diploid), 1 (low-level gain of copy number, generally broad amplifications), and 2 (high-level increase in copy number, often focal amplification). CNV GISTIC 2.0 levels were compiled from the firebrowse (firebrowse.org) data portal (accession date January 15, 2018) for TCGA samples and from the CCLE data portal for cancer cell lines (accession date February 14, 2017).

##### Data Preprocessing and Normalization

The label-free protein quantifications (precursor areas) for COREAD CPTAC samples (16) were first normalized by sample, where summed peak areas for the same protein were divided by the total summed area for the observed sample proteome. Relative protein abundances were then calculated by dividing each protein area over the median area across samples and then log2 transformed. Protein and phosphosite intensities for COREAD cell lines (10) were divided by 100 and transformed to log2. For BRCA cell lines (9), protein log2 fold-changes were calculated by subtracting the median intensities across the samples. Similarly, the label-free protein intensities (peak areas) for BRCA cell lines from (18) were converted into relative abundances by calculating the log2 ratio of protein intensities over the median intensities across samples. Sample replicates of protein and phosphoprotein were combined by averaging the values for each protein and phosphosite, respectively. Phosphopeptides intensities mapping to the same phosphosite were combined by calculating the median phosphosite intensity per sample. In the cancer cell lines, genes with multiple isoforms were filtered by selecting the protein isoform with highest median expression across samples. Proteomics and phosphoproteomics distributions across cancer samples and cell lines were quantile normalized to ensure comparable distributions, using the *normalizeQuantiles* function from Limma R package (24). In total, 13,569 proteins across 436 samples (340 cancer samples and 96 cell lines) and 79,824 phosphosites across 195 samples (145 cancer samples and 50 cell lines) were assembled in this study. Given the sparseness of the phospho(protein) data, for the subsequent analyses, we only selected proteins measured in at least 25% of the 368 samples with protein, mRNA, and CNV measurements and the phosphosites measured in at least 50% of the 170 samples with also phosphorylation data, comprising 8,124 proteins and 5,733 phosphosites. The phospho(protein) and mRNA data were then standardized using the *z*-score transformation.

At the RNA-seq level, lowly expressed genes were removed by filtering out genes with mean counts per million lower than 1 across samples. After raw counts normalization by the trimmed-mean of M-values method (25) using the edgeR R package (26), the log2-counts-per-million values were extracted from the *voom* (27) function in Limma. After merging the CPTAC samples with the CCLE cell lines, the final RNA-seq dataset comprised 13,228 genes with measurements across 370 samples (296 cancer samples and 74 cell lines). At the CNV level, after compiling the GISTIC thresholded data, 19,023 genes were found to have CNV measurements across 412 samples (337 cancer samples and 75 cell lines).

Potential confounding factors revealed by principal component analysis (supplemental Figs. S1*A* and S2*A*) were regressed out using a multiple linear regression model. This model was implemented with the protein or mRNA abundance of a given gene as a dependent variable and the potential confounding factors, *i.e.* cancer type, experimental batch, proteomics technology, age, and gender, as independent variables. The residuals from the linear model were the protein and mRNA variation not driven by the confounding effects, as the second principal component analysis demonstrated (supplemental Figs. S1*B* and S2*B*).

##### Analysis of Protein Attenuation

The strategy in (4) was used to evaluate the impact of CNVs at the genome level on cancer proteomes. For each gene, the Pearson correlation coefficients between the CNV and mRNA and the CNV and protein were calculated, and an attenuation measure devised as follows:

(1) $${\text{Attenuation potential}}_{\text{i}}=\text{corr}\left({\text{CNV}}_{\text{i}},{\text{mRNA}}_{\text{i}}\right)-\text{corr}\left({\text{CNV}}_{\text{i}},{\text{Protein}}_{\text{i}}\right),\text{i}\in \text{Protein}$$

where *corr* represents the Pearson correlation coefficient and *Protein* represents 8,124 genes for which CNV, mRNA, and protein quantifications across 368 samples where available. After calculating the attenuation potentials, a Gaussian mixture model with four mixture components was used to cluster the genes in four different groups. Group 1 had 19 genes with a negative attenuation potential due to the higher correlation between the CNV and protein than with the CNV and mRNA. These genes, which were not attenuated at the protein level, were included with the remaining nonattenuated genes in group 2, comprising 4,689 genes. Groups 3 and 4 contained the lowly attenuated and highly attenuated genes, with 2,578 and 857 genes, respectively. The Gaussian mixture model was implemented using the *Mclust* function from the mclust R package (28).

The enrichment of CORUM complexes was calculated with a hypergeometric model, using the *enrichr* function from the *clusterProfiler* R package (29). Only CORUM complexes with a Jaccard index lower than 0.9 and with more than five proteins were used. The comparison of ubiquitination site fold-changes across protein attenuation levels was done using protein ubiquitination data obtained with three proteasome inhibitors: MG-132, epoxomicin, and bortezomib (30–33).

##### Compendium of Physical Protein Interactions

In order to build a compendium of physical protein interactions, we downloaded a data set of protein–protein interactions from BioGRID version 3.4.157 (34) (accession date January 30, 2018). We only selected protein interactions occurring in human and captured with physical experimental systems. Interactions captured with Affinity Capture-RNA and Protein-RNA were excluded in order to guarantee that our dataset contained only interactions observed at the protein level. After excluding protein homodimers, 524,148 protein interactions (262,074 unique) were compiled with BioGRID. A list of protein interactions was also built using a set of protein complexes from the CORUM database (35) (accession date May 29, 2018). The rationale was that protein partners from the same protein complex interact physically at least once. Using a set of 1,787 protein complexes and excluding protein homodimers, we assembled 74,712 (37,356 unique) physical protein interactions. As small number of 890 Endoplasmic reticulum-related interactions were additionally curated from the literature. In total, 572,856 (286,428 unique) protein physical interactions were compiled.

##### Linear Modeling to Identify Protein and Phospho–protein Associations

##### Protein Associations

For a given protein physical interaction pair X and Y, it was tested whether protein X can control the protein levels of Y through protein–protein interaction, potentially constraining the degradation rate of Y. For each interacting pair, two nested linear models were fitted. The first model (null) was used to predict the protein levels of Y (Py) using its mRNA (Ty) and a set of other covariates, *i.e.* cancer type, experimental batch, proteomics technology, patient age, and gender (Equation (2)). In a second linear model (alternative), the CNV levels of X (Gx) was added as predictor variable (Equation (3)). A likelihood ratio test (LRT) (Equation (4)) was then applied in order to test whether the second model increases the goodness of fit of the first model in predicting Py.

(2) $$\text{Null model}:{\text{P}}_{\text{y}}=\beta_{0}+\beta_{1}{\text{T}}_{\text{y}}+\left[\beta_{2},\beta_{3},\beta_{4},\beta_{5},\beta_{6}\right]+\varepsilon$$

**β**_0_ represents the intercept, **β**_1_ the regression coefficient (effect size) for the mRNA of Y, **β**_2_, **β**_3_, **β**_4_, **β**_5,_ and **β**_6_ the regression coefficients for the covariates cancer type, experimental batch, proteomics technology, age, and gender, respectively. ε is the noise term.

(3) $$\text{Alternative model}:{\text{P}}_{\text{y}}=\beta_{0}+\beta_{1}{\text{T}}_{\text{y}}+\left[\beta_{2},\beta_{3},\beta_{4},\beta_{5},\beta_{6}\right]+\beta_{7}{\text{G}}_{\text{x}}+\varepsilon$$

**β**_7_ is the regression coefficient for the CNV (G_x_) of protein X. An LRT was used to assess the significance of the association:

(4) $$\text{LRT}=2\times \left[{\text{log}}_{\text{e}}\text{Lik}\left(\text{Alternative}\right)-{\text{log}}_{\text{e}}\text{Lik}\left(\text{Null}\right)\right]$$

log_e_Lik corresponds to the log likelihood of the alternative and null models. *p* values were then calculated using the LRT statistic over a chi-squared distribution and adjusted for false discovery rate (FDR) using the Benjamini–Hochberg method. This model was applied for a given protein association pair X and Y if: X ϵ CNV Λ Y ϵ Protein Λ Y ϵ mRNA, where CNV, Protein, and mRNA are the sets of genes detected with the respective assays.

A total of 411,591 protein pairs followed these criteria and were tested across 368 tumor samples. The same analysis was performed with the mRNA, instead of the CNV, of protein X for 392,128 protein pairs. To avoid spurious protein associations that might occur due to the genomic co-localization of the controlling proteins, the top-ranked association was selected using the Borda ranking method. This was done systematically for every case where multiple controlling proteins in the same chromosome were associated with the same controlled protein. More than one controlling protein in the same chromosome for the same controlled protein were allowed if their CNV profile Pearson correlation was lower than 0.5.

The linear models were implemented using *lm* R function and the LRT test with associated statistics were calculated using the *lrtest* function from the lmtest R package. The Borda ranking method was implemented using the *Borda* function from the TopKLists R package (36).

##### Phospho–protein Associations

For a given protein pair X and Y, it was tested whether a phosphosite Xp from protein X can be associated with changes in the protein abundance of protein Y. A similar model to before linear regression models and LRT tests was used. For each phosphosite–protein interaction, a first null model was fitted to predict the protein levels of Y (Py) using its mRNA (Ty), the CNV and protein levels of protein X (Gx and Px), and the covariates experimental batch, patient age, and gender (Equation (5)). In a second alternative linear model, the phosphosite Xp (Phox) of protein X was added as predictor variable (Equation (6)). The models were then compared using an LRT as in Equation (4).

(5) $$\text{Null model}:{\text{P}}_{\text{y}}=\beta_{0}+\beta_{1}{\text{T}}_{\text{y}}+\beta_{2}{\text{G}}_{\text{x}}+\beta_{3}{\text{P}}_{\text{x}}+\left[\beta_{4},\beta_{5},\beta_{6}\right]+\varepsilon$$

where **β**_0_ represents the intercept, **β**_1_ the coefficient of the mRNA of Y, **β**_2_ and **β**_3_ the regression coefficients for the CNV and protein of X, respectively, and **β**_4_, **β**_5,_ and **β**_6_ the regression coefficients for the covariates experimental batch, age, and gender, respectively. ε is the noise term.

(6) $$\text{Alternative model}:{\text{P}}_{\text{y}}=\beta_{0}+\beta_{1}{\text{T}}_{\text{y}}+\beta_{2}{\text{G}}_{\text{x}}+\beta_{3}{\text{P}}_{\text{x}}+\left[\beta_{4},\beta_{5},\beta_{6}\right]+\varepsilon$$

where **β**_7_ is the regression coefficient for the phosphosite Xp of protein X. This model was applied for a given phosphosite–protein association pair Xp and Y if Xp ϵ Phospho Λ X ϵ Protein Λ X ϵ CNV Λ Y ϵ Protein Λ Y ϵ mRNA, where Phospho, Protein, CNV, and mRNA are the sets of genes detected with the respective assays. A total of 315,772 phosphosite–protein pairs followed this criteria and were tested with this model across 170 tumor samples.

##### Structural Analysis

Protein interface sizes were calculated using an in-house pipeline (int3dInterfaces, github.com/evocellnet/int3dInterfaces) that extracts protein interfaces from Interactome3D structures (37). For each protein interaction structure in Interactome3D, this pipeline uses NACCESS (bioinf.manchester.ac.uk/naccess) to calculate the solvent accessibility of the bound and unbound monomers. Every residue changing its relative solvent accessibility is considered to form part of the interface. From the 11,530 human protein interaction structures analyzed with this pipeline, structures of protein homodimers or structures with less than 100 amino acids were removed. Also, structures with chain lengths bigger than the respective UniProt protein lengths and with the same chain length for each partner were removed. After applying these filters, 3,082 structures with 6,147 protein interactions were used in the subsequent analyses.

For the 1,470 proteins that contained both information about CNV attenuation and interface size, the percentage of residues in protein interfaces was calculated as the ratio of the number of unique residues in interfaces over the protein size. For 60 significant protein association pairs represented in the structural data, the relation between the protein interface size with the regression CNV coefficient and FDR, was assessed using the Pearson correlation coefficient. For each pair, the protein interface size was calculated in the controlling and controlled proteins. The protein sizes (number of residues) were obtained from UniProt for 20,349 proteins (accession date June 19, 2018).

The percentage of area inside complex for the protein subunits from the COP9 signalosome was calculated using FreeSASA (38). For each protein subunit, this percentage corresponded to the difference between the solvent accessible surface area (SASA) outside and inside complex over the SASA outside complex. The SASA was calculated in units of squared Ångström (Å^2^).

##### Analysis of Gene Essentiality Using CRISPR-Cas9 screenings

Gene essentiality data obtained with CRISPR-Cas9 screenings (39) were downloaded from Project Achilles data portal (portals.broadinstitute.org/Achilles) (accession date October 31, 2017). These data contain gene-dependence levels adjusted for copy-number-specific effects for 17,670 genes across 341 cancer cell lines. Genes with an essentiality score lower than −1×S.D. (the standard deviation for the entire dataset corresponds to 0.3) in more than 5% of the cell lines were considered essential and used in the remaining analysis (5,532 genes). The median gene essentiality was calculated for 3,548 genes with attenuation and essentiality data across the 341 cancer cell lines.

##### Pairwise Correlation of Protein Association Pairs Using Normal Tissue Data

Gene and protein expression data for normal human tissues were obtained from the Genotype-Tissue Expression (GTEx) (40) and Human Proteome Map (41) portals. The gene expression was obtained in the format of RNA-seq median Reads Per Kilobase of transcript per Million mapped reads for 56,238 genes across 53 tissues. The protein expression was downloaded as averaged label-free spectral counts for 17,294 genes across 30 tissues. For the protein expression data, 9,156 genes in common with the Human Proteome Map data available in Expression Atlas (42) were selected. The 14 tissues common to the GTEx and the Human Proteome Map used in the remaining analysis were: frontal cortex, spinal cord, liver, ovary, testis, lung, adrenal gland, pancreas, kidney, urinary bladder, prostate gland, heart, esophagus, and colon. The gene expression in the last three tissues was averaged in GTEx, between heart atrial appendage and left ventricle; between esophagus gastroesophageal junction, mucosa, and muscularis; and between colon sigmoid and transverse. The protein and gene expression data were then filtered to only include genes and proteins expressed in at least 10 of 14 tissues, resulting in 5,239 genes consistently expressed at the gene and protein level. The RNA and protein measurements were then standardized to *z*-scores and quantile normalized.

Having assembled the gene and protein expression datasets for normal tissues, pairwise Pearson correlation coefficients were calculated between the protein of the controlling and controlled genes, mRNA of the controlling and controlled genes, and mRNA and protein of the controlled gene. The Pearson correlations were calculated for 91 highly significant associations (FDR < 0.01), 210 significant associations (0.01 ≤ FDR < 0.05), and 161,945 nonsignificant associations at the CNV and mRNA level (FDR ≥ 0.05). In order to assure that the increase in protein–protein correlations were not simply due to an increase in mRNA–mRNA correlations, we selected the protein pairs with mRNA Pearson's correlation coefficient between 0 and 0.4, corresponding to 57,145 pairs (30 highly significant, 69 significant and 57,046 nonsignificant).

##### Analysis of the Impact of CNV Attenuation on the eQTL Association to Disease Traits

Following the approach in HipSci proteomics (43), we considered a stringent set of 21,601 associations from the NHGRI-EBI GWAS catalogue (download on April 10, 2018; converted to hg19) for analysis. We considered eQTLs reported from the GTEx in 35 tissues (excluding brain), compute the number of tissues having the same slope sign, *i.e.* direction of effect size, and discarded those with consistent slope in less than three tissues.

We defined proxy variants of each *cis*-eQTL as variants in high LD (r^2^ > 0.8; based on the UK10K European reference panel) within the same *cis* window. Next we grouped eQTLs in high LD blocks (r^2^ > 0.8), excluded from this analysis 247 genes having each more than 100 eQTL blocks) and obtain a final set of 66,197 eQTL blocks corresponding to 2,953 genes and 441,194 eQTL–gene associations. We then define these blocks as GWAS tagging if for at least one eQTL in the block at least one LD proxy variant was annotated in the NHGRI-EBI GWAS catalogue. Finally, we report the fraction of GWAS-tagging eQTL stratified by the attenuation level of the corresponding *cis* genes. To assess the robustness of this analysis and to study the effects on GWAS-tagging probability of eQTL recurrence across tissues, we compute the number of tissues in which an eQTL was called with the same slope and report results by stratifying the eQTLs by increasing number of tissues.

We rely on core protein complexes from CORUM to identify the gene complex membership status and segregate those that are annotated in at least one large complex (>5 subunits). Out of the genes with eQTL evidence and with annotation scores, 961 are annotated in CORUM, and 576 are members of large complexes.

## RESULTS

### 

#### 

##### Protein-level Attenuation of Gene Dosage Associates with Distinct Essentiality and Structural Features

**Fig. 1.**
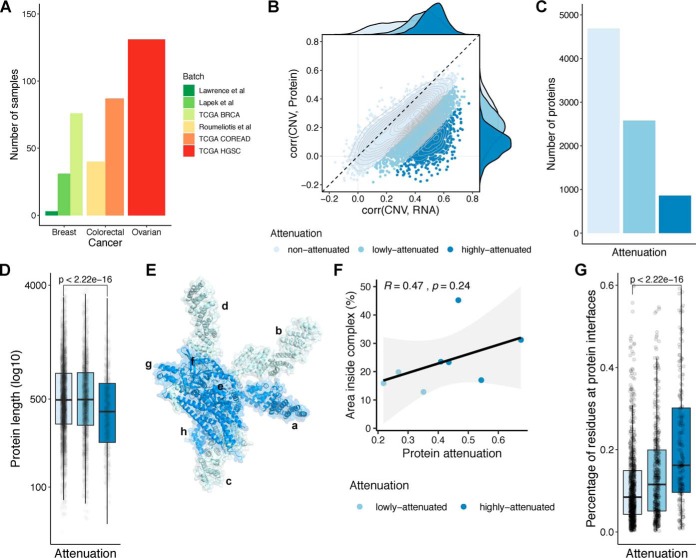
**Features of proteins showing gene dosage buffering at the protein level.** (*A*) Number of samples with CNV, mRNA, and protein measurements, by cancer type and batch. (*B*) Scatter plot representing the correlation between the CNV and mRNA (*x*-axis) and the CNV and protein (*y*-axis), for each gene. The colors represent the attenuation levels. From light blue to dark blue: nonattenuated, low attenuated and highly attenuated. (*C*) Number of proteins by attenuation level. (*D*) Protein length (log10 of number of residues) by attenuation level. (*E*) Representation of COP9 signalosome complex. (*F*) Scatter plot representing the correlation between the attenuation potential (*x*-axis) and the fraction of residues at interfaces in the complex (*y*-axis), for the eight protein subunits from the COP9 signalosome complex represented in (*E*). (*G*) Percentage of residues at protein interfaces by attenuation level.

In order to study protein posttranscriptional control, we collected matched gene copy number, mRNA, and protein expression cancer datasets made available by the TCGA and CPTAC consortia, for BRCA (15, 44), ovarian (17, 45), and COREAD cancers (16, 46). In addition, we compiled existing protein/gene expression and copy-number data for cancer cell lines from Lape*k et al.* (BRCA) (9), Roumeliotis *et al.* (COREAD) (10), and Lawrence *et al.* (BRCA) (18). In total, 368 cancer samples (294 tumors and 74 cell lines) were compiled in our study with matched gene expression, copy number, and protein abundance (Fig. 1*A*). Principal component analysis revealed the presence of confounding effects in the RNA and protein expression data (Figs. S1*A* and S2*A*). These effects are related to cancer type, experimental batch, type of proteomics experiment, and also patient gender and age. Therefore, these potential confounding effects were regressed out from the RNA and protein expression data (“Methods”). After correction, the association between the principal components and the potential confounding effects was removed (Figs. S1*B* and S2*B*). In the combined dataset, the average mRNA–protein correlation is 0.44, which is in agreement with previous studies.

We then investigated the impact of CNV in cancer proteomes, using the strategy reported in Gonçalves *et al.* (4). Due to the sparseness of the protein data, we selected genes with protein measurements in at least 25% of the 368 samples, comprising 8,124 genes with CNV, mRNA, and protein expression. We included the CNV, mRNA and protein measurements for the 8,124 genes in the Supplemental Table 1. For each gene, we then calculated the Pearson correlation coefficient between the CNV and the mRNA and the CNV and the protein, across samples. In order to assess the disagreement between the transcriptome and proteome regarding the copy number changes, we calculated an attenuation potential, corresponding to the difference between Pearson coefficients (“Methods”). A higher attenuation potential suggests genes that have CNVs buffered at the protein level. As previously mentioned, we then clustered the genes by attenuation potential using an unsupervised Gaussian mixture model. Using this strategy, we identified 3,435 (42%) genes as attenuated at the protein level (2,578 low attenuated and 857 high attenuated) and 4,689 as nonattenuated (Figs. 1*B* and 1*C* and Supplemental Table 2). These results indicate that up to 42% of genes show signs of gene dosage buffering at the protein level, probably due to a posttranscriptional control of protein degradation, and robustly recapitulates previous findings on a smaller set of 6,418 genes (4). To discard the possibility that the lack of correlation between CNV and protein could be due to noisiness in measuring protein levels, we asked if the correlation of protein or mRNA measurements, across samples, could predict known protein–protein associations from CORUM, among protein pairs at different levels of attenuation (Fig. S3). As can be seen by the receiver operating curves, protein interaction pairs are better predicted by protein than mRNA correlations in all classes, with the difference increasing with the attenuation level. If the attenuation was mostly explained by noise in the protein-level measurements, then the opposite trend would be expected, where correlation of protein measurements would be noisier for highly attenuated proteins and a worse predictor of protein–protein interactions. These results indicate that attenuated genes are not defined as such because they have noisier protein measurements.

In line with previous findings, the list of attenuated genes is strongly enriched in well-characterized protein complex members and, notably, in members of large complexes (Fig. S4). More, the attenuation potential is correlated with the number of subunits in a protein complex, indicating that members of large complexes have higher attenuation than those of small complexes (Fig. S4*E*). Attenuated genes are also expected to show increased ubiquitination after proteasome inhibition, which was confirmed here using previously published data with three different proteasome inhibitors—MG-132, epoxomicin, and bortezomib (30–33) (Fig. S5*A*). Having defined a comprehensive list of genes/proteins with different degrees of attenuation, we then set out to characterize their physical and genetic properties.

We first asked if the level of attenuation relates to distinct essentiality features, based on gene essentiality defined by CRISPR-Cas9 screens (39). Highly attenuated proteins showed higher gene essentiality than low- and nonattenuated proteins (Fig. S5*B*) (Wilcoxon rank-sum test *p* value < 2.2e-16, highly *versus* nonattenuated proteins). This result is likely to be driven by the enrichment of protein complex members of essential complexes, such as the ribosome and spliceosome. We then studied the physical characteristics of these proteins, such as length and structural properties. We found that the highly attenuated proteins tend to have a smaller size (Fig. 1*D*) (Wilcoxon rank-sum test *p* value < 2.2e-16; highly- *versus* nonattenuated proteins), suggesting a size-dependent buffering mechanism. For the structural analysis, we considered a total of 2,392 proteins having structurally defined interface models (37). We illustrate this analysis with the COP9 signalosome complex (Fig. 1*E*) where we noticed a trend in which the subunits with a larger surface buried in interfaces had the strongest attenuation (Fig. 1*F*). While the trend on a single complex is not significant (Fig. 1*F*), this trend was supported across all proteins, with the average fraction of residues at interfaces increasing from the nonattenuated to the highly attenuated proteins in a statistically significant manner (Fig. 1*G*).

##### Protein Interaction-dependent Control of Degradation Depends on Interface Size

The features of highly attenuated proteins suggest that protein interactions are an important determinant of a protein's susceptibility of having gene dosage attenuation. It has been suggested that some members of protein complexes can act as scaffolding or rate-limiting subunits. We have previously analyzed a set of 58,627 protein interactions among complexes curated in CORUM database and identified a set of 48 interactions in which a protein can indirectly control the abundance of an interacting partner (4). Here we set out to expand this analysis to all currently reported human physical interactions in the BioGRID database (“Methods”). In total, we collected 572,856 physical interactions and identified proteins whose CNV changes correlate with the protein abundance of interacting proteins once their mRNA levels are taken into account (“Methods”). For an interaction pair of proteins X and Y, we used a linear regression model, where we predict the protein levels of protein Y using the CNV of X, discounting the mRNA of Y and the impact of other covariates (“Methods”). Correlating molecular changes with DNA variation such as CNVs ensures the correlations found are most likely causal and in the direction of DNA changes to the molecular changes. Copy-number alterations in cancer most often occur in large segments leading to co-amplification or co-deletion of multiple co-localized genes. For proteins with two or more interacting partners that are genomically co-localized, we selected only the top-ranking association to avoid spurious “passenger” associations (“Methods”).

**Fig. 2.**
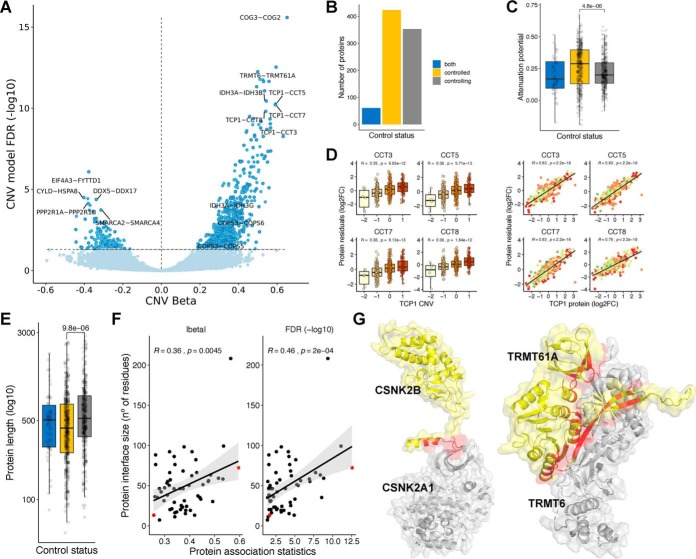
**Physical protein associations.** (*A*) Volcano plot of CNV beta (*x*-axis) and FDR (*y*-axis) for 411,591 protein pairs. Nonsignificant associations (FDR > 5%) are represented in light blue, and significant associations (FDR < 5%) in dark blue. Associations also found to be significant (FDR < 5%) in the mRNA model and filtered by genomic co-localization are highlighted with a darker border (516). (*B*) Number of proteins by control status. (*C*) Distribution of attenuation potential by control status. (*D*) Examples of protein associations between TCP1 (*controlling* protein) and CCT3, CCT5, CCT7, and CCT8 (controlled proteins). The boxplots show the relation between the CNV changes of TCP1 and the protein residuals (log2FC) of the interacting partners. The scatter plots show the same relation with the protein abundance of TCP1. (*E*) Protein length (log10 of number of residues) by control status. (*F*) Scatter plots displaying the correlation between the protein association statistics (beta and FDR) with the protein interface size (number of residues at the protein interface, measured in the controlled protein). Each dot is a protein association. Two representative associations between CSNK2A1 - CSNK2B (small interface) and TRMT6 - TRMT61A (big interface) are denoted in red. (G) Representation of protein interactions between CSNK2A1 and CSNK2B and TRMT6 and TRMT61A. The controlled proteins are colored in yellow (CSNK2B and TRMT61A), and the controlling proteins are colored in gray (CSNK2A1 and TRMT6). The interface area is represented in red.

Out of 572,856 physical interaction,s we had data to test associations for 411,591 with this model, finding 516 protein–protein associations as significant using CNV and mRNA (FDR < 5%) (Fig. 2*A* and Supplemental Table 3). In this set of associations, we classified the proteins as *controlling* (353)—those capable of controlling the protein levels of their interactions partners; *controlled* (423)—whose abundance levels depends on their interactions; and *both* (60)—proteins with the two characteristics (Fig. 2*B*). Out of 423 controlling proteins, 62 had at least two interactions. The top controlling protein was TCP1, which was predicted to control the protein abundance of seven complex partners, including CCT3, CCT5, CCT7, and CCT8 (Fig. 2*D*). As expected, the controlled proteins had higher attenuation potential, a consequence of the posttranscriptional regulation of their protein levels (Fig. 2*C*) (Wilcoxon rank-sum test *p* value < 4.8e-6; controlled versus controlling proteins). The controlled proteins also show a smaller size (Wilcoxon rank-sum test *p* value < 9.8e-6; controlled versus controlling proteins), which corroborates the hypothesis that protein size is important for the buffering mechanism (Fig. 2*E*). These results increased the evidence of interactions and regulators that may act as drivers of protein complex assembly.

We hypothesized that protein-interaction-dependent control of degradation could depend on the protein interfaces size. To test this, we identified 60 significant associations with available structural models (“Methods”) and correlated the protein interface size with the effect size (beta value) and significance (FDR) of the respective protein association pairs (Fig. 2*F*). We found that both statistics are positively and significantly correlated with interface size (CNV beta - Pearson's r: 0.36; *p* value: 4.5e-3; -log10 FDR - Pearson's r: 0.46; *p* value: 2.0e-4). We selected two examples to illustrate the observed differences (Fig. 2*G*). Posttranscriptional regulation of TRMT61A by TRMT6, that form the tRNA (adenine-N1-)-methyltransferase enzyme, is the second-strongest association found in our analysis, and the interface formed between these two proteins covers a total of 72 residues. In contrast, a weaker association between CSNK2A1 and CSNK2B may be explainable by a much smaller interface of 13 residues.

These results show that interface sizes are an important determinant of the protein-interaction-mediated control of protein degradation. This may be due to an effect of binding affinity or differences in the recognition of exposed interfaces of different sizes by the degradation machinery.

##### Identification of Phosphorylation Sites That May Modulate Protein Complex Assembly

**Fig. 3.**
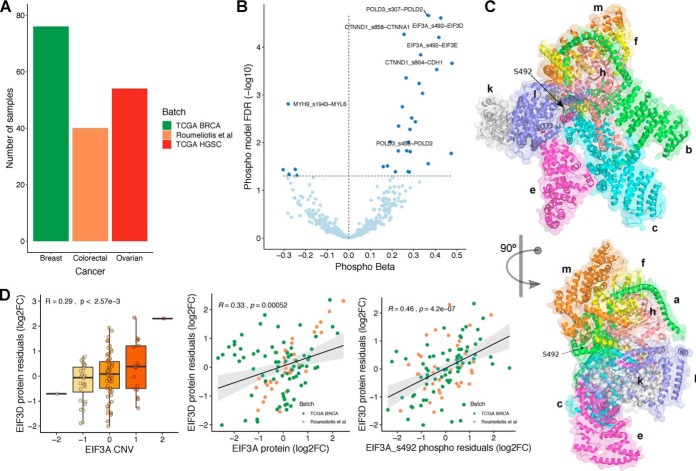
**Identification of phosphorylation sites with a potential role in regulating protein interactions.** (*A*) Number of samples with CNV, mRNA and phospho(protein) measurements, by cancer type/batch. (*B*) Volcano plot of phospho beta (*x*-axis) and FDR (*y*-axis). Each dot is a phosphosite–protein association, between a putative regulatory phosphosite Xp and a regulated protein Y. All associations (438) are significant in the CNV and mRNA models between the putative regulatory protein X and the regulated protein Y. 32 associations (FDR < 5%) are also significant in the phospho model (dark blue). (*C*) Representation of EIF3 complex in two orientations. The arrow points to the phosphosite S492 (serine 492) at EIF3A subunit. (*D*) Significant association between EIF3A/EIF3A S492 and EIF3D. The boxplots show the agreement between the CNV changes of EIF3A and the protein residuals (log2FC) of EIF3D. The scatter plots show the same relation with the protein and phosphosite (S492) abundances of EIF3A.

The role of phosphorylation in modulating protein-binding affinities has been well described (47–49). We reasoned we could use the multi-omics datasets to find protein interactions affected by phosphorylation, which in turn could impact complex assembly and protein degradation. Out of 368 samples with CNV, mRNA, and protein measurements, 170 also have quantifications at the phosphosite level (Fig. 3*A*). For this analysis, we used proteins and phosphosites measured in at least 50% of the 170 samples, corresponding to 8,546 proteins and 5,733 phosphosites.

Using the compendium of physical interactions (572,856 protein interactions), we tested whether the changes of a phosphosite Xp from protein X is associated with the protein levels of the interacting protein Y. As before, we used a linear regression model where the protein abundance of protein Y is predicted using the phosphosite levels of protein X (Xp), while taking into account the protein and CNV levels of protein X, the RNA of protein Y, and other covariates (“Methods”). Out of 315,772 phosphosite–protein pairs tested with this model, 11,672 associations were significant (FDR < 5%). To ensure the associations are directional, we overlapped these associations with the 516 protein–protein associations found with the CNV and mRNA models, identifying 32 overlapping associations (Fig. 3*B*, listed in Supplemental Table 4). Our interpretation of these associations is that these phosphosites can regulate the protein interaction and thereby modulate the degradation of the complex subunits.

The 32 associations involve 28 phosphosites, and of these, two phosphosites are already known to regulate interactions (POLD3 S458 and MYH9 S1943) and an additional case (EIF3A S492) is not yet known to regulate protein interactions but is at the interface with other complex members (Fig. 3*C)*. EIF3A is predicted here to be a “rate-limiting” subunit of the eukaryotic initiation factor 3 complex and has been previously experimentally implicated in the control of protein levels of several of the other subunits (50). One phosphosite of EIF3A (S492) showed a strong association with the protein levels of two other complex subunits (EIF3D and EIF3E). In line with this, we find that the copy number of EIF3A correlates with the residual protein levels of EIF3D (*i.e.* after regressing out EIF3D mRNA levels) and that the phosphosite levels of EIF3A S492 correlates better with EIF3D protein residual than the EIF3A total protein levels (Fig. 3*D*). We further confirmed that the residual phosphosite levels of EIF3A S492 phosphosite (once accounting for the protein abundance of EIF3A) is significantly correlated with the protein levels of EIF3D in both datasets analyzed (Fig. S6). However, we found dataset-specific differences in the correlation of the protein levels of EIF3A and EIF3D (Fig. S6). Overall, these results suggest that EIF3A S492 may have an impact on protein complex assembly.

##### Protein Attenuation Mechanisms Found in Cancer Are Observed in Normal Tissues

The study of the impact of CNVs in cancer proteomes indicates that up to ∼40% of genes have copy number changes that are buffered at the protein level. Such posttranscriptional regulatory processes should not be specific to cancer; however, the extent that these effects are observed in normal cellular states is still largely unknown. To address this question, we analyzed gene and protein expression datasets for normal tissues, made available by the GTEx and Human Protein Map projects. In total, we collected expression for 5,239 proteins and genes, across 14 tissue types (“Methods”).

**Fig. 4.**
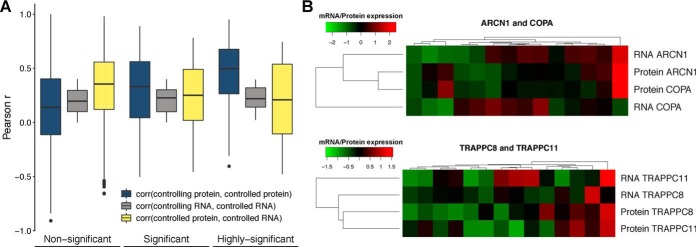
**Evidence of interaction-mediated control of protein abundances in normal tissues.** (*A*) Pearson correlation coefficient between the protein of the controlling and controlled genes (blue); mRNA of the controlling and controlled genes (gray) and mRNA and protein abundance of the controlled gene (yellow); for the nonsignificant associations (FDR > 5%), significant associations (1% < FDR < 5%) and highly significant associations (FDR < 1%). (*B*) Heatmap showing the agreement between the mRNA and protein expression profiles (rows) across tissues (columns) for two highly significant associations: ARCN1 (controlling) ∼ COPA (controlled) and TRAPPC8 (controlling) ∼ TRAPPC11 (controlled).

We tested if the posttranscriptional control dependent on protein interactions observed in cancer is present in normal tissues. For this, we asked if the protein abundance of controlling–controlled protein pairs will tend to correlate more strongly than other protein interaction pairs. Similarly, we expected that the correlation between the mRNA and protein levels of controlled subunits would tend to be weaker than for non-posttranscriptionally controlled proteins. We tested this using protein–protein interaction pairs measured in the tissue data with significant controlling–controlled relationships from cancer data (301 pairs) and all other 161,945 protein–protein interactions pairs (“Methods”). Reassuringly, we observed that the correlation of protein abundance across tissues increased for protein pairs with stronger association strength, for similar levels of mRNA-mRNA correlation values (Wilcoxon rank-sum test *p* value = 8.96e-4 between nonsignificant and significant pairs; *p* value = 8.25e-06 between nonsignificant and highly significant pairs) (Fig. 4*A*). Also, as predicted the protein to mRNA correlation values across tissues, of the controlled subunits, decreases with the association strength (Wilcoxon rank-sum test *p* value = 0.022 between nonsignificant and significant pairs) (Fig. 4*A*). We provide two examples for protein-interacting pairs ARCN1 and COPA and TRAPPC8 and TRAPPC11 where the mRNA levels of the controlling subunits (ARCN1 and TRAPPC8) appear to dictate the protein abundance of both proteins (Fig. 4*B*). These results suggest that the protein associations identified in the cancer datasets can also be observed in normal tissues, at least in aggregate. Importantly, they demonstrate that cancer data can be a useful resource to study protein homeostasis in normal conditions.

##### Buffering of Gene Expression Variation Due to Natural Genetic Variation

**Fig. 5.**
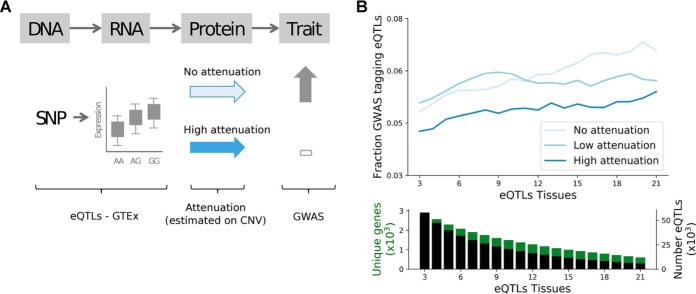
**Protein attenuation reduces cis-eQTLs impact on phenotypic traits.** (*A*) Illustration of the potential impact of protein attenuation on the eQTL association with phenotypic changes. (*B*) Fraction of eQTLs associated to disease traits for the three classes of CNV attenuation: no (light blue), low (blue), and high (dark blue) attenuation. The fractions are reported for increasing number of eQTL tissues, *i.e.* minimal number of tissues in which an eQTL was called (*x*-axis). *Bottom panel* shows the number of genes and eQTLs used in the top figure for cumulative strata of eQTL tissues.

If mechanisms controlling the protein levels are consistent across cell types, then the attenuation models studied here could help elucidate how natural variation may sometimes result in changes in mRNA but not protein and consequently phenotypic traits. Single-nucleotide polymorphisms associated with gene expression via quantitative trait loci (QTL) analysis—known as expression QTLs (eQTLs)—should also tend to be attenuated at protein level potentially for the same genes as those found in cancer. To study this, we analyzed if protein-level CNV buffering could explain the probability of eQTLs to have phenotypic impact, *i.e.* in high LD (r^2^ > 0.8) with GWAS variants (Fig. 5*A* and “Methods”; on genes with significant CNV-mRNA Pearson's r > 0.3). To this end, we relied on *cis*-eQTLs reported in GTEx and compared the fraction of GWAS tagging eQTLs for different classes of protein attenuation (Fig. 5*B* and “Methods”). We found that eQTLs corresponding to genes classified as highly attenuated have a lower fraction of GWAS tagging eQTLs, and that the difference between the degree of attenuation increases for eQTLs mapped in multiple tissues (Fig. 5*B*).

Highly attenuated genes tend to be enriched in protein complexes and are likely essential to the cell, and therefore could have specific biases as to how eQTLs are linked to GWAS associated traits. To account for this potential bias we replicated the analysis on members of protein complexes. Interestingly, this shows that the attenuation score has a higher impact on GWAS tagging probability for members of protein complexes, and more specifically for members of large protein complexes (>5 subunits) (Fig. S7).

These results suggest that the CNV attenuation measured in cancer cells for protein abundance has direct application in the ranking of potential impact of mRNA variation on phenotype differences and support the idea that some of these attenuation mechanisms may take place in multiple tissues.

## DISCUSSION

The joint analysis of multi-omics datasets of cancer samples suggests that a very significant fraction of the proteome (up to 42%) is under posttranscriptional control. The set of genes with protein-level buffering of CNVs is enriched in gene products belonging to large protein complexes. In addition, we found that the fraction of interface residues of a protein is a strong determinant of attenuation. Together with experiments on pulse-chase degradation (6), aneuploidy (1–3), and the impact of natural genetic variation on protein levels (51, 52), these results implicate protein complex formation as an important factor in posttranscriptional control, most likely via a high degradation rate of unassembled subunits. We note that this mechanism of CNV buffering at the protein level may be possible with CNV amplifications and deletions. While in the former it would be manifested by an apparent increase in the degradation rate of free complex subunits, in the latter it would result from a decrease in the apparent degradation rate of free subunits. However, it is likely that multiple mechanisms contribute to posttranscriptional control measured in the cancer samples including, for example, the control of protein translation rates by microRNAs or RNA-binding proteins. The extent of posttranscriptional control that is explained by the different processes remains to be studied.

We observed that the fraction of residues at the interface correlates with the probability that a protein shows gene dosage attenuation. Similarly, the size of the interface correlates with the strength of association between pairs of physical interactions in which one subunit appears to control the abundance level of the interaction partner. The size of the interface typically correlates with increasing binding affinity between proteins as well as larger amounts of hydrophobic residues that are exposed in the absence of interactions. We speculate that either of these consequences could play a role in the attenuation. In particular, larger fraction of hydrophobic regions could increase the propensity to form aggregates, and in some cases hydrophobic regions are known to be recognized for degradation (53). This could represent a general mechanism for recognition of unassembled complex subunits. The structural analysis performed here is limited by the current lack of coverage for structures of protein complexes. In the future, additional structures may allow us to study in more detail the interface features that are important for the attenuation mechanism.

We have used data from cancer samples to identify the attenuated proteins and physical interactions with rate-limiting subunits. We find that most of the controlling–controlled protein–protein associations we predict have a positive relationship. Given the working model that these are explained by protein complex formation, the negative associations could be explained by cases of mutually exclusive complex membership. The fact that few associations predicted are negative are consistent with the idea that most complex members are not mutually exclusive.

It is still unclear if the same proteins and interactions will have the same posttranscriptional control in other systems and/or species. When studying expression variation in normal tissues and the association of eQTLs with phenotypes, we observed that, in aggregate, the same proteins and interactions show signals consistent with posttranscriptional buffering of mRNA expression variation. Of note, we find that eQTLs are less likely to be linked to phenotypes in highly attenuated proteins. This is in line with studies of mRNA and protein QTLs in human induced pluripotent stem cell lines, showing that genetic variation driving mRNA changes are more likely to be associated to genotype differences when they are observed at the protein level (43). These findings highlight the importance of studying the degree of conservation of these posttranscriptional processes in different tissues and systems in the context of human genetics and disease.

## Supplementary Material

Supplementary Figures and Legends

Supplementary Table 1

Supplementary Table 3

Supplementary Table 4

Supplementary Table 2
