# Supplementary material for: Multi-omics Characterization of Interaction-mediated Control of Human Protein Abundance levels
Source: Mol Cell Proteomics. 2019 Jun 25;18(8 Suppl 1):S114–25. doi: 10.1074/mcp.RA118.001280 (PMC6692786; doi:10.1074/mcp.RA118.001280)
Supplement: Supplementary Figures and Legends [file 142565_1_supp_345415_psvvh7.pdf]

Multi-omics characterization of interaction-mediated control of human protein abundance levels

Abel Sousa[1,2,3,4], Emanuel Gonçalves[5], Bogdan Mirauta[4], David Ochoa[4], Oliver Stegle[4,6,7], Pedro Beltrao[4,#]

[1]Instituto de Investigação e Inovação em Saúde da Universidade do Porto (i3s), Rua Alfredo Allen 208, 4200-135, Porto, Portugal
[2]Institute of Molecular Pathology and Immunology of the University of Porto (IPATIMUP), Rua Júlio Amaral de Carvalho 45, 4200-135, Porto, Portugal
[3]Graduate Program in Areas of Basic and Applied Biology (GABBA), Abel Salazar Biomedical Sciences Institute, University of Porto, Rua de Jorge Viterbo Ferreira 228, 4050-313, Porto, Portugal
[4]European Molecular Biology Laboratory, European Bioinformatics Institute, Wellcome Genome Campus, Hinxton, CB10 1SD, Cambridge, UK
[5]Wellcome Sanger Institute, Wellcome Genome Campus, Hinxton, UK
[6]European Molecular Biology Laboratory, Genome Biology Unit, 69117 Heidelberg, Germany
[7]Division of Computational Genomics and Systems Genetics, German Cancer Research Center (DKFZ), 69120, Heidelberg, Germany
[#]Correspondence to: pbeltrao@ebi.ac.uk

Supplementary Figure and Table Legends

Supplementary figure 1. Confounding effects regressed-out from transcriptomics data. (A) Pearson correlation coefficient of the first 10 principal components (PCs) with the potential confounding effects before normalization. **(B)** Pearson correlation coefficient of the first 10 principal components (PCs) with the potential confounding effects after normalization.

Supplementary figure 2. Confounding effects regressed-out from proteomics data. (A) Pearson correlation coefficient of the first 10 principal components (PCs) with the potential confounding effects before normalization. **(B)** Pearson correlation coefficient of the first 10 principal components (PCs) with the potential confounding effects after normalization.

Supplementary figure 3. ROC curve analysis for the prediction of true protein-protein interacting pairs. Correlation of protein abundance and gene expression were used as a predictor of CORUM protein pairs, among the highly-attenuated **(A)** lowly-attenuated **(B)** and non-attenuated **(C)** protein pairs. The x-axis represents the false-positive rate (FPR) and the y-axis the true-positive rate (TPR). The AUC of each curve is indicated.

Supplementary figure 4. Relationship between CNV attenuation in proteins and protein complex membership. (A) List of top protein complexes ordered by enrichment in attenuated genes. X-axis shows p-values derived with an hypergeometric test (Benjamini-Hochberg multiple test correction). We discarded from the list of CORUM complexes those with a Jaccard index higher than 0.9 with any other complex and those with 5 proteins or less **(B)** Number of genes for each attenuation class by complex membership status (CORUM). **(C)** Number of genes stratified by the maximum number of subunits of any protein complex incorporating the genes. **(D)** Enrichment of attenuated proteins in members of protein complexes stratified by complex size, i.e number of subunits. Shown are the p-values derived with an exact Fisher test (alternative "greater"). **(E)** Relationship between the number of subunits in a protein complex and the protein complex member CNV attenuation. For each member of a complex we juxtapose the attenuation score (x-axis) and the maximum number of subunits of any complex this protein is part of (y-axis).

Supplementary figure 5. Attenuated proteins show faster increase in protein ubiquitination after proteasome inhibition and higher gene essentiality. (A) Ubiquitination sites fold-changes (y-axis) across protein attenuation levels (x-axis) after proteasome inhibition with three inhibitors: Bortezomib, Epoxomicin and MG-132. **(B)** Median gene essentiality measured in CRISPR-Cas9 screenings (y-axis), across 341 cancer cell lines, by attenuation level (x-axis).

Supplementary figure 6. Correlation of EIF3A protein and EIF3A_s492 phosphosite with EIF3D protein. The scatterplots show the Pearson's correlation coefficient between EIF3A protein log2FC **(A)** and EIF3A_s492 phosphosite log2FC **(B)** with EIF3D protein residuals (after regressing-out the mRNA and possible confounding factors from protein expression). The EIF3A protein abundance and confounding factors were also regressed-out from EIF3A_s492 phosphorylation levels. The correlations are shown with all samples and by dataset (TCGA breast cancer samples (BRCA) and colorectal cancer cell lines from Roumeliotis et al).

Supplementary figure 7. Impact of the CNV attenuation at protein level on the eQTL association with disease traits stratified by the protein complex membership status. Identical analysis as in figure 3D is performed for genes with no (A) and with (B) existing annotation in CORUM protein complexes, and (C) for genes members of protein complexes with at least 5 subunits. Bottom panels: the number of genes and eQTLs considered for the analysis shown in the top panels.

Supplementary Tables

Supplementary table 1. CNV, mRNA and protein measurements across cancer samples for 8,124 genes. The CNV data is represented as discretized GISTIC scores (**Methods**). The mRNA and protein measurements are represented as z-scores, with potential confounding factors regressed-out using a multiple linear regression model (**Methods**). The cancer type (breast, colorectal, ovarian), experimental batch (TCGA BRCA, TCGA COREAD, TCGA HGSC, Lawrence et al, Roumeliotis et al or Lapek et al) and proteomics type (TMT or label-free) of each sample are also included as columns.

Supplementary table 2. 8,124 genes stratified by attenuation level. The table includes the Pearson correlation coefficient between the CNV and mRNA and the CNV and protein, respective p-values and attenuation potential.

Supplementary table 3. 516 protein-protein associations significant in the CNV and mRNA models (FDR < 5%). For each association, the table includes the controlling and controlled protein, and the effect size (beta) and FDR from both models.

Supplementary table 4. 32 significant phospho-protein associations (FDR < 5%). The table includes the controlling protein/phosphosite, the controlled protein, and the effect size (beta) and FDR from the phospho model. All associations are also significant in the CNV and RNA models, between the putative regulatory and regulated proteins.

Figure S1

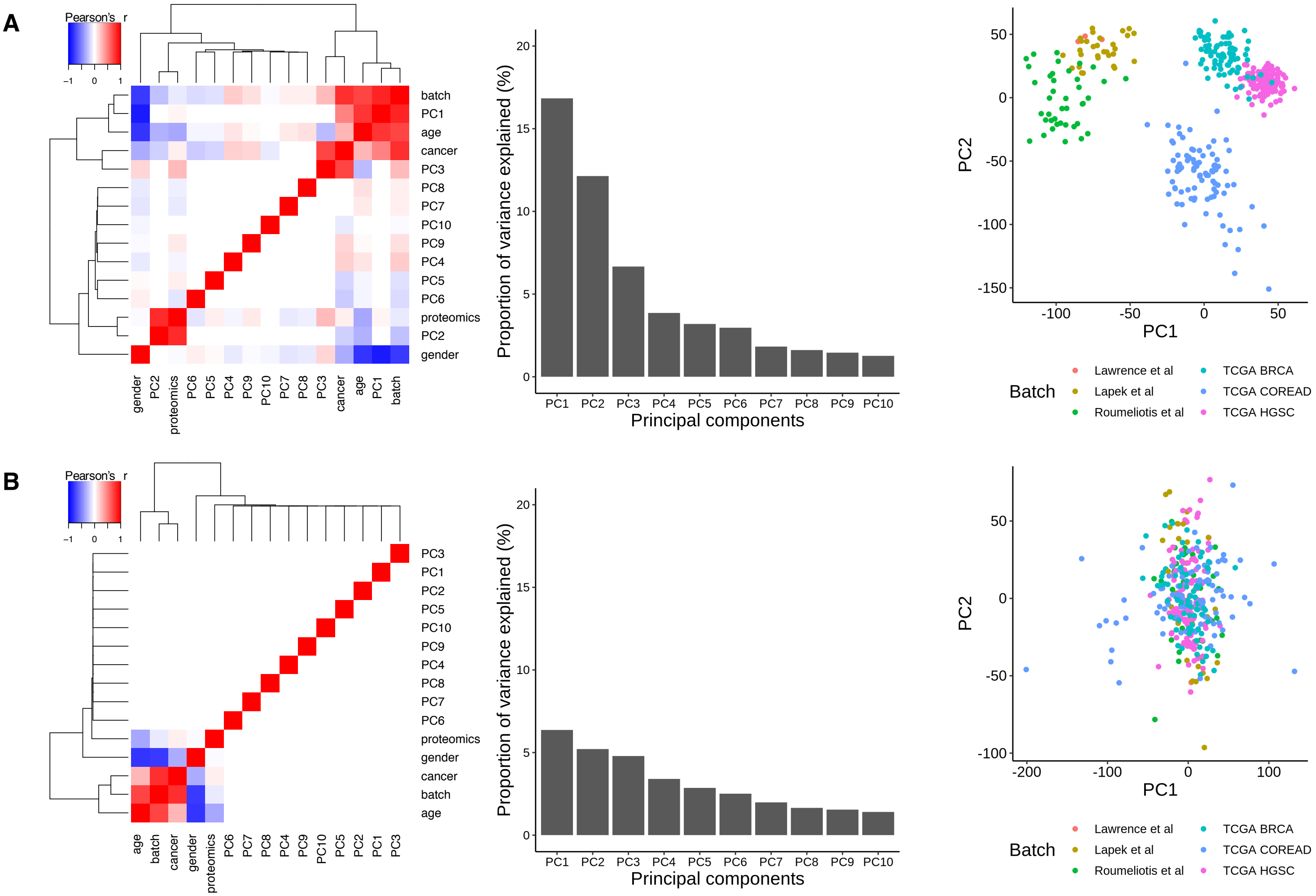
A
Pearson's r
−1 0 1
batch
PC1
age
cancer
PC3
PC8
PC7
PC10
PC9
PC4
PC5
PC6
proteomics
PC2
gender
Proportion of variance explained (%)
Principal components
PC2
PC1
Batch
Lawrence et al
Lapek et al
Roumeliotis et al
TCGA BRCA
TCGA COREAD
TCGA HGSC
B
Pearson's r
−1 0 1
PC3
PC1
PC2
PC5
PC10
PC9
PC4
PC8
PC7
PC6
proteomics
gender
cancer
batch
age
Proportion of variance explained (%)
Principal components
PC2
PC1
Batch
Lawrence et al
Lapek et al
Roumeliotis et al
TCGA BRCA
TCGA COREAD
TCGA HGSC

Figure S2

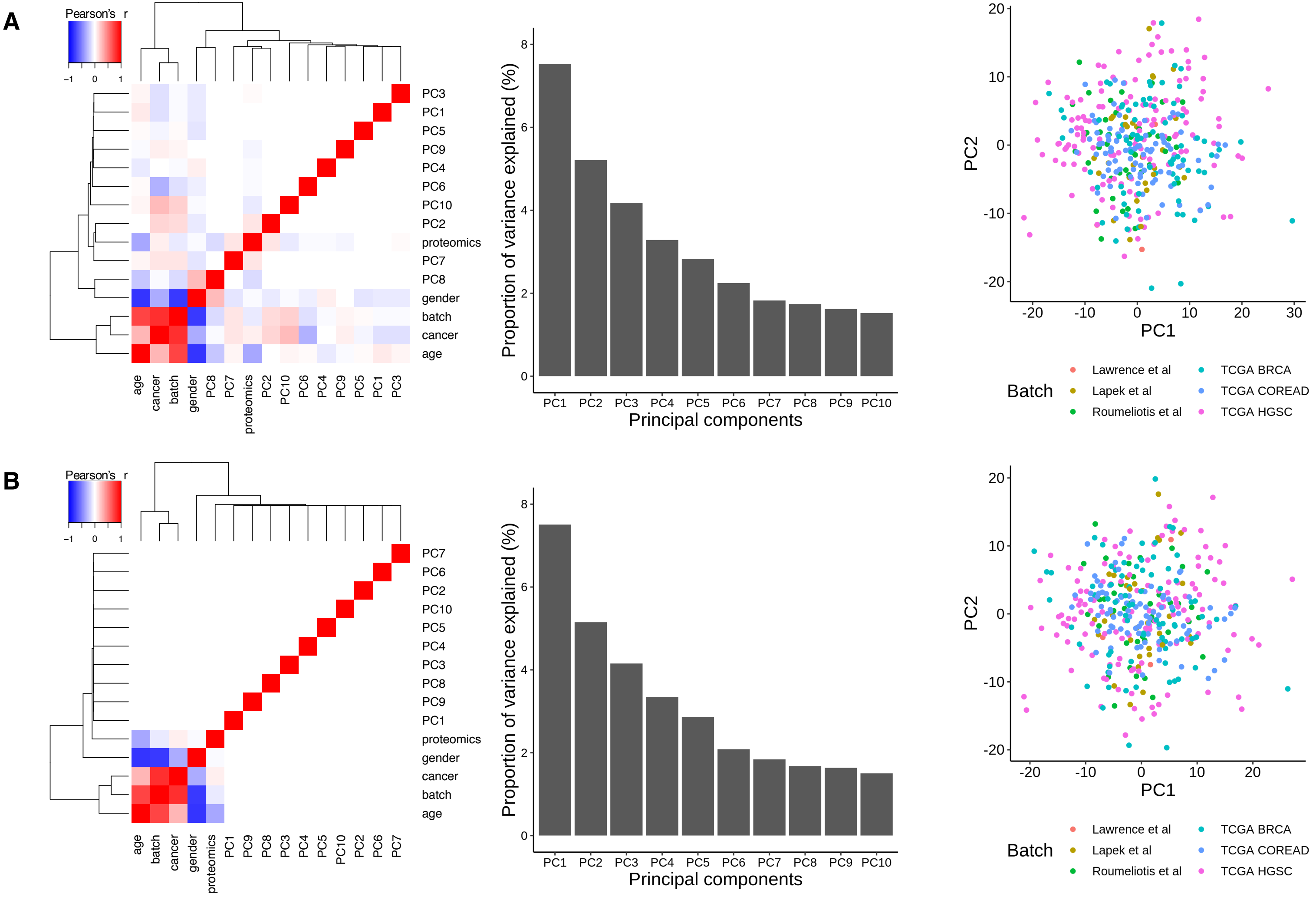
A
Pearson's r
-1
0
1
PC3
PC1
PC5
PC9
PC4
PC6
PC10
PC2
proteomics
PC7
PC8
gender
batch
cancer
age
Proportion of variance explained (%)
Principal components
PC2
PC1
Batch
Lawrence et al
Lapek et al
Roumeliotis et al
TCGA BRCA
TCGA COREAD
TCGA HGSC
B
Pearson's r
PC7
PC6
PC2
PC10
PC5
PC4
PC3
PC8
PC9
PC1
proteomics
gender
cancer
batch
age
Proportion of variance explained (%)
Principal components
PC2
PC1
Batch
Lawrence et al
Lapek et al
Roumeliotis et al
TCGA BRCA
TCGA COREAD
TCGA HGSC

Figure S3

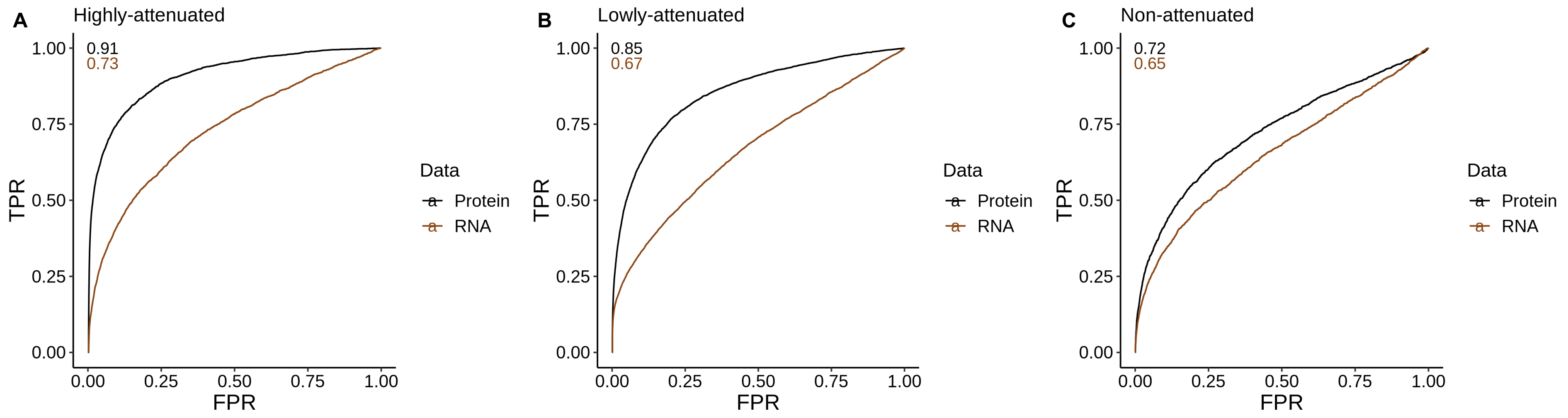
A
Highly-attenuated
0.91
0.73
TPR
FPR
0.00
0.25
0.50
0.75
1.00
Data
Protein
RNA
B
Lowly-attenuated
0.85
0.67
C
Non-attenuated
0.72
0.65

Figure S4

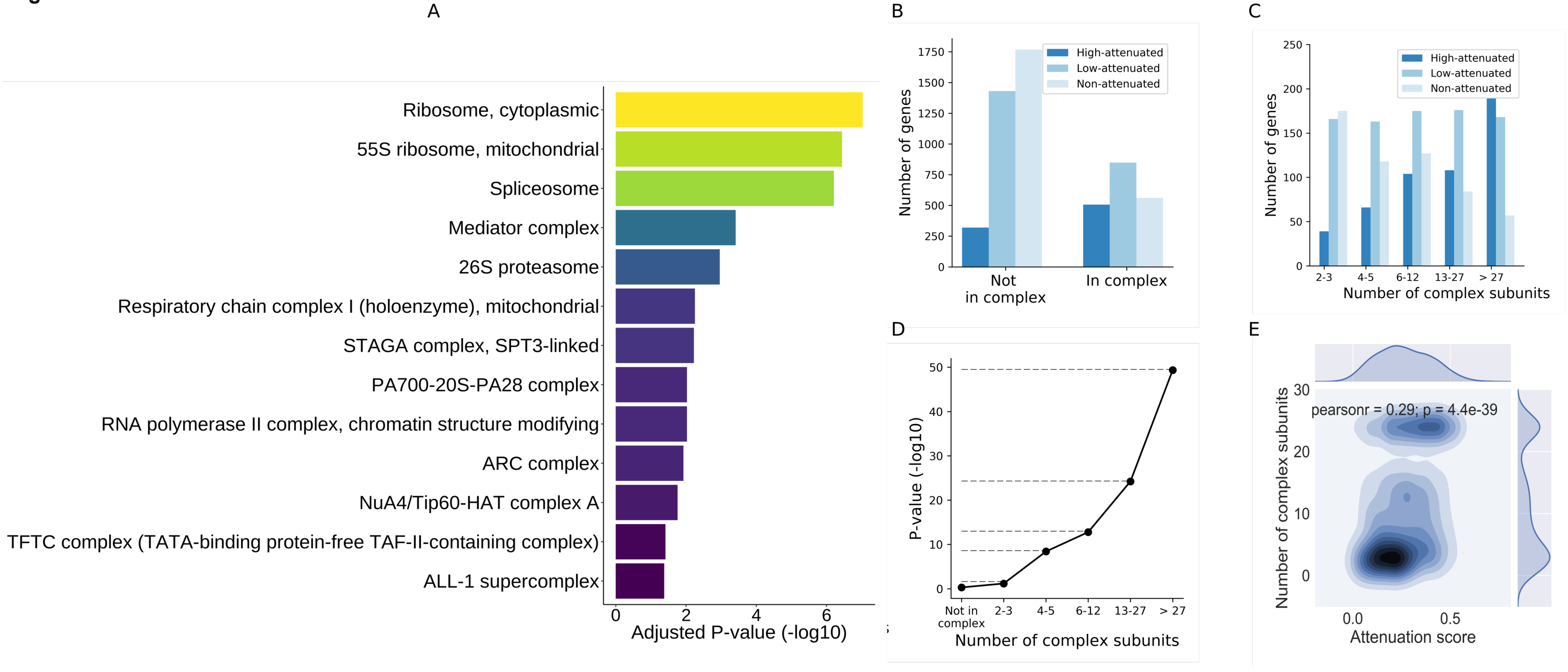
A
Ribosome, cytoplasmic
55S ribosome, mitochondrial
Spliceosome
Mediator complex
26S proteasome
Respiratory chain complex I (holoenzyme), mitochondrial
STAGA complex, SPT3-linked
PA700-20S-PA28 complex
RNA polymerase II complex, chromatin structure modifying
ARC complex
NuA4/Tip60-HAT complex A
TFTC complex (TATA-binding protein-free TAF-II-containing complex)
ALL-1 supercomplex
0
2
4
6
Adjusted P-value (-log10)
B
Number of genes
High-attenuated
Low-attenuated
Non-attenuated
Not in complex
In complex
C
Number of genes
High-attenuated
Low-attenuated
Non-attenuated
2-3
4-5
6-12
13-27
> 27
Number of complex subunits
D
P-value (-log10)
Not in complex
2-3
4-5
6-12
13-27
> 27
Number of complex subunits
E
pearsonr = 0.29; p = 4.4e-39
Number of complex subunits
Attenuation score

Figure S5

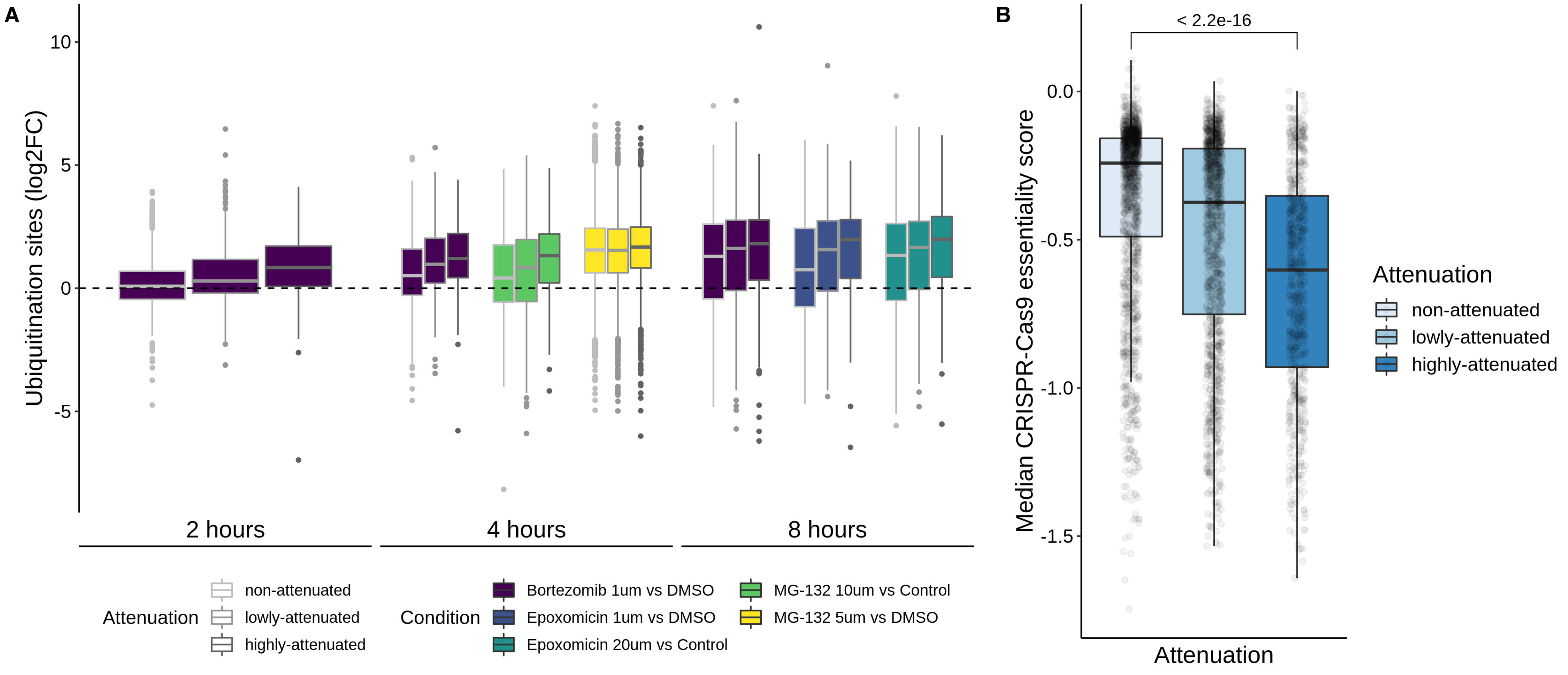
A
B
Ubiquitination sites (log2FC)
10
5
0
-5
2 hours
4 hours
8 hours
Attenuation
non-attenuated
lowly-attenuated
highly-attenuated
Condition
Bortezomib 1um vs DMSO
Epoxomicin 1um vs DMSO
Epoxomicin 20um vs Control
MG-132 10um vs Control
MG-132 5um vs DMSO
< 2.2e-16
Median CRISPR-Cas9 essentiality score
0.0
-0.5
-1.0
-1.5
Attenuation
Attenuation
non-attenuated
lowly-attenuated
highly-attenuated

Figure S6

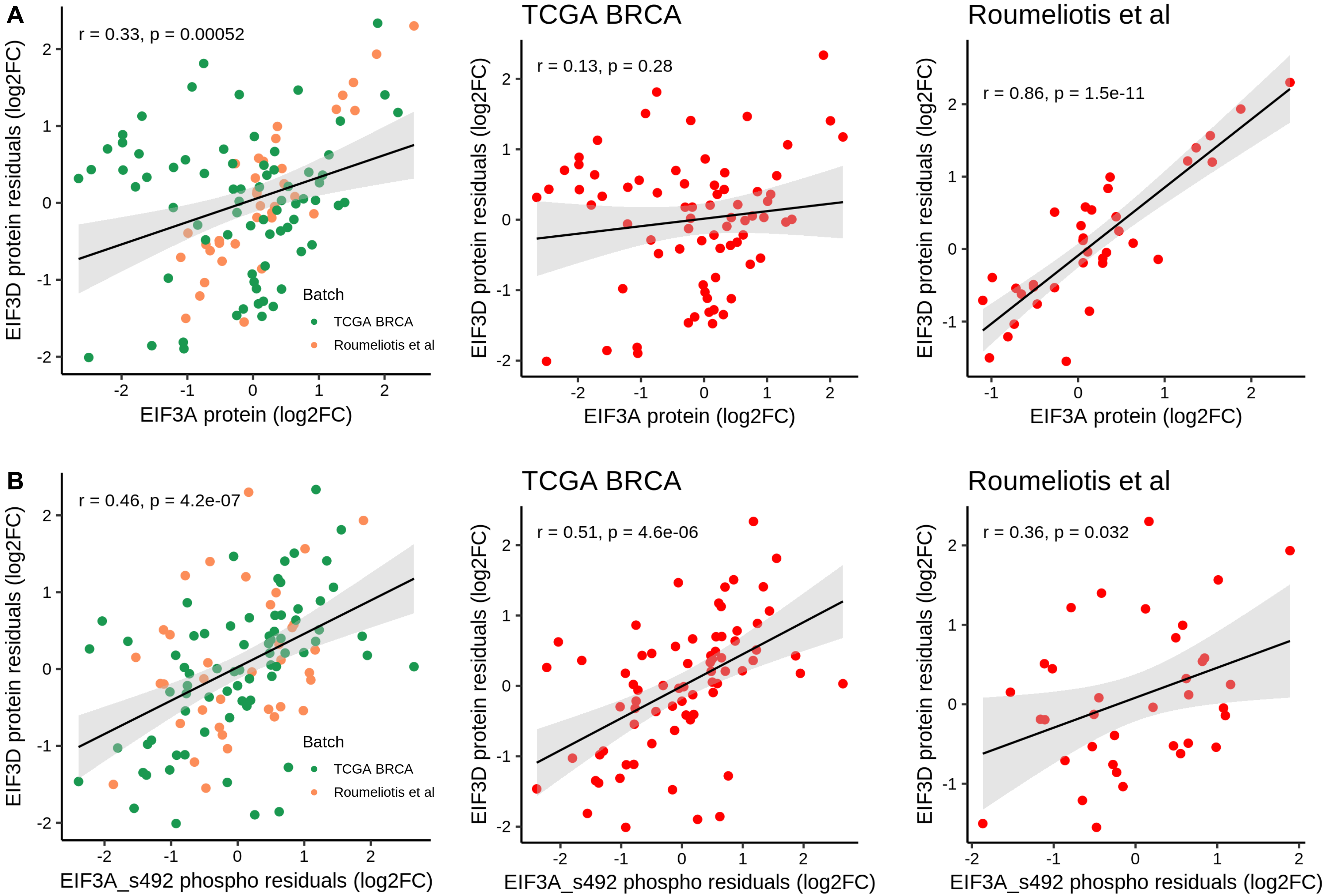
A
r = 0.33, p = 0.00052
EIF3D protein residuals (log2FC)
EIF3A protein (log2FC)
Batch
TCGA BRCA
Roumeliotis et al
TCGA BRCA
r = 0.13, p = 0.28
Roumeliotis et al
r = 0.86, p = 1.5e-11
B
r = 0.46, p = 4.2e-07
EIF3A_s492 phospho residuals (log2FC)
r = 0.51, p = 4.6e-06
r = 0.36, p = 0.032

Figure S7

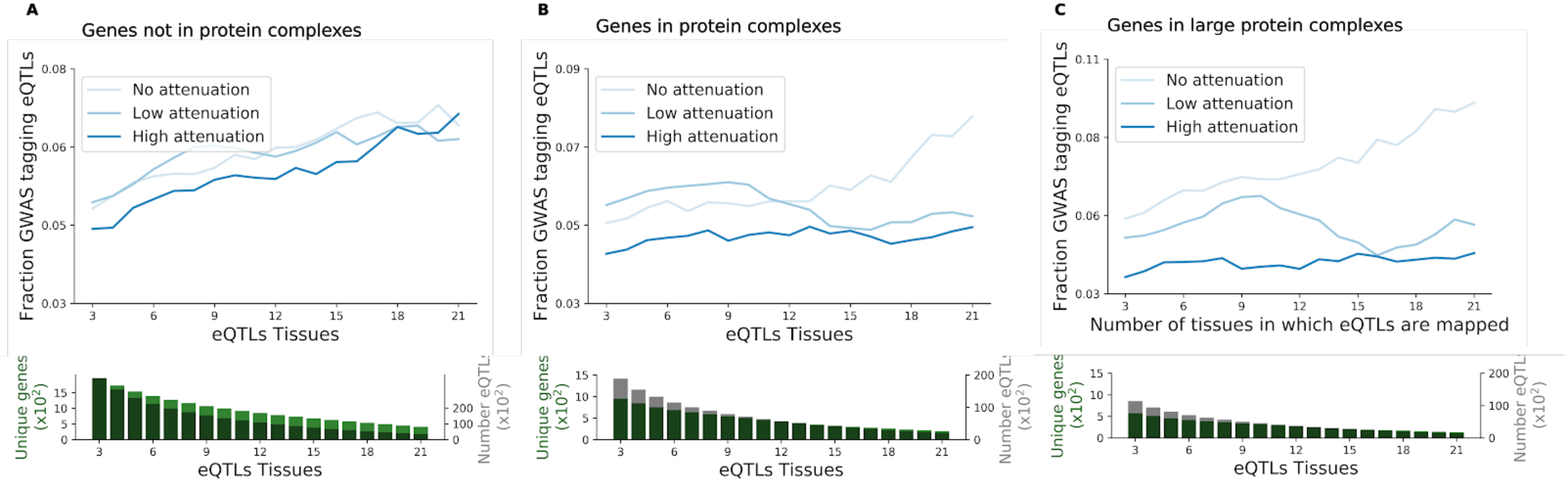
A
Genes not in protein complexes
No attenuation
Low attenuation
High attenuation
Fraction GWAS tagging eQTLs
eQTLs Tissues
Unique genes (x10^2)
Number eQTLs (x10^2)
B
Genes in protein complexes
No attenuation
Low attenuation
High attenuation
Fraction GWAS tagging eQTLs
eQTLs Tissues
Unique genes (x10^2)
Number eQTLs (x10^2)
C
Genes in large protein complexes
No attenuation
Low attenuation
High attenuation
Fraction GWAS tagging eQTLs
Number of tissues in which eQTLs are mapped
Unique genes (x10^2)
Number eQTLs (x10^2)
eQTLs Tissues
